# A novel prognostic score model incorporating CDGSH iron sulfur domain2 (CISD2) predicts risk of disease progression in laryngeal squamous cell carcinoma

**DOI:** 10.18632/oncotarget.8150

**Published:** 2016-03-17

**Authors:** Lin Yang, Shaodong Hong, Yan Wang, Zhenyu He, Shaobo Liang, Haiyang Chen, Shasha He, Shu Wu, Libing Song, Yong Chen

**Affiliations:** ^1^ Department of Radiation Oncology, Sun Yat-Sen University Cancer Center, Guangzhou 510060, China; ^2^ State Key Laboratory of Oncology in Southern China, Guangzhou 510060, China; ^3^ Collaborative Innovation Center for Cancer Medicine, Guangzhou 510060, China; ^4^ The First Hospital of Foshan, Foshan 528000, China; ^5^ The Sixth Affiliated Hospital of Sun Yat-sen University, Guangzhou 510060, China

**Keywords:** CDGSH iron sulfur domain2 (CISD2), laryngeal squamous cell carcinoma (LSCC), progression free survival (PFS), biomarker

## Abstract

**Background:**

The role of CDGSH iron sulfur domain 2 (CISD2) in laryngeal squamous cell carcinoma (LSCC) remains unclear.

**Results:**

CISD2 were up-regulated in LSCC tissues compared with adjacent noncancerous tissues both at mRNA and protein levels. CISD2 was significantly correlated with T stage, lymph node metastasis, clinical stage and disease progression. A prognostic model (C-N model) for PFS was subsequently constructed based on independent prognostic factors including CISD2 and N classification. This model significantly divided LSCC patients into three risk subgroups and was more accurate than the prediction efficacy of TNM classification in the training cohort (C-index, 0.710 vs 0.602, *P* = 0.027) and validation cohort (C-index, 0.719 vs 0.578, *P* = 0.014).

**Methods:**

Real-time PCR and Western blotting were employed to examine the expression of CISD2 in eight fresh paired LSCC samples. Immunohistochemistry was performed to assess CISD2 expression in 490 paraffin-embedded archived LSCC samples. A prognostic model for progression-free survival (PFS) was built using independent factors. The concordance index (C-Index) was used to evaluate the prognostic ability of the model.

**Conclusions:**

CISD2 was up-regulated in LSCC. The novel C-N model, which includes CISD2 levels and N classification, is more accurate than conventional TNM classification for predicting PFS in LSCC.

## INTRODUCTION

Laryngeal squamous cell carcinoma (LSCC) is the second most common head and neck squamous cell carcinoma worldwide and accounts for about 2.4% of all newly diagnosed malignancies worldwide each year [1]. It is epidemiologically related to smoking and the male to female ratio of LSCC is 6:1 [2]. Surgery plus postoperative radiotherapy, or chemoradiotherapy was the primary treatment option to preserve physical functions such as swallowing, respiration and voicing of laryngeal [3]. Although there have been significant improvements in terms of therapeutic regimens, the loco-regional recurrence rates of LSCC patients, especially those with advanced stages remains challenging [4, 5]. In clinical practice, treatment decision-making and prediction of clinical outcomes for LSCC patients is mainly based on TNM staging system, which inevitably ignores the impact of tumor biology and is not accurate enough [6]. Previous studies have revealed that the genetic background also has prognostic value for head and neck cancers [7]. There is undoubtedly urgency for a novel prognostic model that utilizes both biomarkers and clinical classification to identify those patients with a poor prognosis before treatment to make sure the treatment of LSCC patients is more individualized.

The CDGSH iron sulfur domain2 (CISD2) is an evolutionarily conserved gene that is located within the region on human chromosome 4q24 [8]. CISD2 is a member of iron sulfur proteins, forming a homodimer harboring two redox-active 2Fe–2S clusters. It is primarily located at the endoplasmic reticulum or mitochondrial membranes [9, 10]. Transcriptional splicing error of CISD2 in mice leads to breakdown and dysfunction of mitochondria, which is an causative of the neurological genetic disorder Wolfram Syndrome (WFS) [11]. CISD2 is primarily involved in the regulation of calcium (Ca^2+^) homeostasis, autophagy and apoptosis by interacting with B cell lymphoma 2 (BCL-2) [12]. Furthermore, CISD2 also participate in the regulation of cellular iron and active oxygen species (ROS) homeostasis and hence is critical in the process of cancer cell proliferation and tumor progression [13]. Recent studies have demonstrated CISD2 is elevated in human breast cancer and early-stage cervical cancer [14, 15]. However, little is known about the expression and clinical significance of CISD2 in LSCC.

In this study, we assessed the expression of CISD2 in a series of LSCC specimens and investigated its associations with clinicopathological parameters and prognostic value in LSCC patients. We constructed a novel prognostic score model combining the CISD2 and the N stage to better predict the prognosis of LSCC patients, which was more accurate than the prediction efficacy of TNM classification alone. The results indicate that CISD2 may serve as a prognostic factor and potentially a therapeutic target.

## RESULTS

### Patient clinical characteristics

A total of 245 and 245 patients from the training and the validation cohorts were included for analyses. Median follow-up for PFS in the training cohort and the validation cohort were 55.6 months and 55.0 months, respectively. Five-year events rates for PFS in the training and validation cohorts were 67.3% and 70.2%, respectively. Details of patient characteristics are shown in Table 1.

**Table 1:** Association between CISD2 expression and the clinicopathological features of the training cohort and validation cohort with LSCC patients by Pearson's χ^2^ and Fisher's exact tests

Feature	Total	Training cohort			Validation cohort		
		Low expression (%)	High expression (%)	*P*	Low expression (%)	High expression (%)	
Total	490	127	118		120	125	
Age (years)				0.065			0.349
< 60	253 (51.6%)	60 (49.6%)	61 (50.4%)		61 (46.2%)	71 (53.8%)	
≥ 60	237 (48.4%)	67 (54.0%)	57 (46.0%)		59 (52.2%)	54 (47.8%)	
Gender				*0.486			*1.000
Male	481 (98.2%)	125 (51.7%)	117 (48.3%)		117 (49.0%)	122 (51.0%)	
Female	9 (1.8%)	2 (66.7%)	1 (33.3%)		3 (50.0%)	3 (50.0%)	
Smoking status				0.260			0.498
Absent	66 (13.5%)	19 (61.3%)	12 (38.7%)		19 (54.3%)	16 (45.7%)	
Present	424 (86.5%)	108 (50.5%)	106 (49.5%)		101 (48.1%)	109 (51.9%)	
Drinking status				0.851			0.036
Absent	297 (60.6%)	76 (51.4%)	72 (48.6%)		81 (54.4%)	68 (45.6%)	
Present	193 (39.4%)	51 (52.6%)	46 (47.4%)		39 (40.6%)	57 (59.4%)	
Pathological differentiation				0.624			0.584
Highly	196 (40.0%)	54 (52.9%)	48 (47.1%)		48 (51.1%)	46 (48.9%)	
Moderately	228 (46.5%)	58 (53.2%)	51 (46.8%)		59 (49.6%)	60 (50.4%)	
Poorly	66 (13.5%)	15 (44.1%)	19 (55.9%)		13 (40.6%)	19 (59.4%)	
Site				0.214			0.125
Glottic	198 (40.4%)	44 (46.8%)	50 (53.2%)		45 (43.3%)	59 (56.7%)	
Non-Glottic	292 (59.6%)	83 (55.0%)	68 (45.0%)		75 (53.2%)	66 (46.8%)	
T classification				< 0.001			< 0.001
1+2	235 (48.0%)	92 (67.6%)	44 (32.4%)		70 (70.7%)	29 (29.3%)	
3	133 (27.1%)	21 (36.2%)	37 (63.8%)		26 (34.7%)	49 (65.3%)	
4	122 (24.9%)	14 (27.5%)	37 (72.5%)		24 (33.8%)	47 (66.2%)	
N classification				0.047			< 0.001
0	379 (77.3%)	106 (55.5%)	85 (44.5%)		107 (56.9%)	81 (43.1%)	
1	64 (13.1%)	15 (45.5%)	18 (54.5%)		7 (22.6%)	24 (77.4%)	
2+3	47 (9.6%)	6 (28.6%)	15 (71.4%)		6 (23.1%)	20 (76.9%)	
Clinical stage				< 0.001			< 0.001
I	211 (43.1%)	77 (66.4%)	39 (33.6%)		68 (71.6%)	27 (28.4%)	
II	118 (24.1%)	25 (44.6%)	31 (55.4%)		23 (37.1%)	39 (62.9%)	
III	161 (32.9%)	25 (34.2%)	48 (65.8%)		29 (33.0%)	59 (67.0%)	
IV							
Treatment method				< 0.001			0.228
Surgery	339 (69.2%)	96 (64.4%)	53 (35.6%)		97 (51.1%)	93 (48.9%)	
Comprehensive Treatment	151 (30.8%)	31 (32.3%)	65 (67.7%)		23 (41.8%)	32 (58.2%)	
Progression				< 0.001			< 0.001
No	337 (68.8%)	104 (63.0%)	61 (37.0%)		98 (57.0%)	74 (43.0%)	
Yes	153 (31.2%)	23 (28.8%)	57 (71.2%)		22 (30.1%)	51 (69.9%)	

Abbreviations: LSCC, laryngeal Squamous cell carcinoma (LSCC); Highly, Highly differentiated; Moderately, Moderately differentiated; Poorly, Poorly differentiated; Non-Glottic, Supraglottic and Subglottic; Comprehensive Treatment, Surgery and chemotherapy or Surgery and radiotherapy; CISD2, CDGSH iron sulfur domain2; *represent the results from the Fisher's exact test.

### CISD2 is overexpressed in human SCC

**Figure 1:**
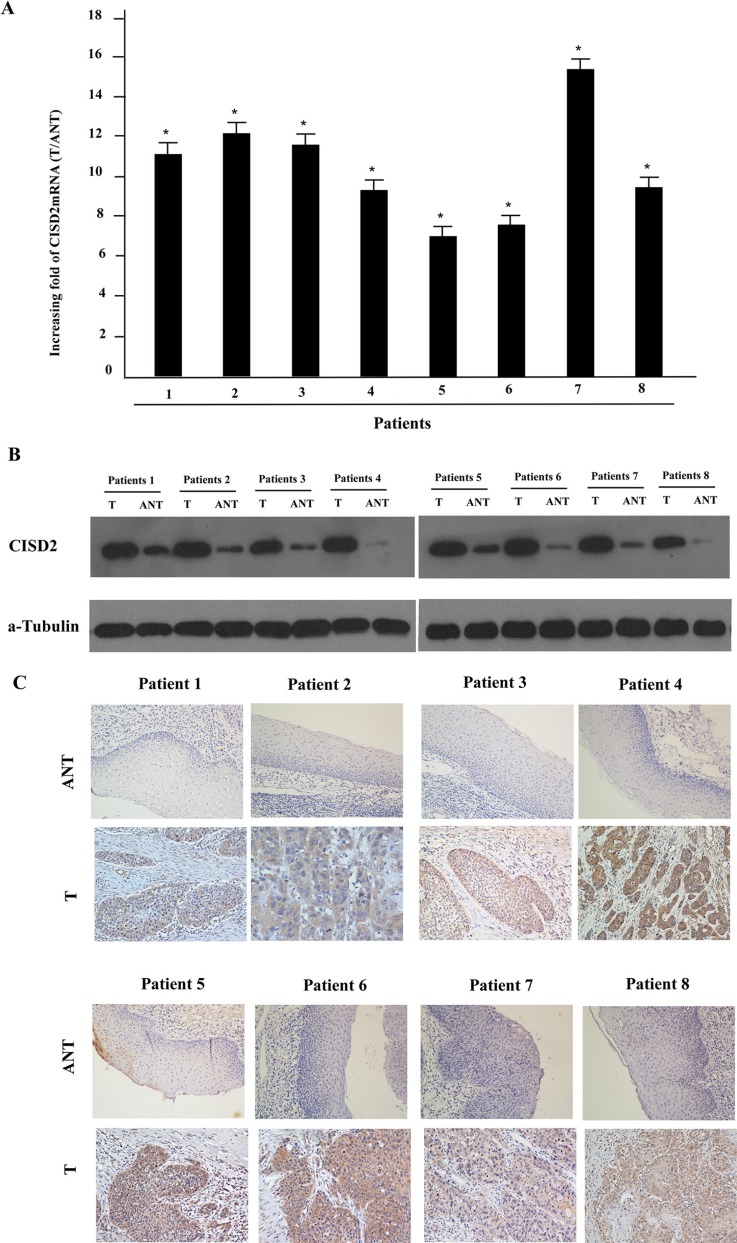
Overexpression of CISD2 mRNA and protein in human laryngeal squamous cell carcinoma (LSCC) tissues (**A**) CISD2 mRNA expression in eight matched pairs of LSCC tissues (T) and adjacent noncancerous tissues (ANT), as quantified by qPCR and normalized to the expression of *GAPDH*. Error bars are the standard deviation of the mean (SD) for three experiments performed in parallel. (**B**) Representative western blotting analyses of CISD2 protein expression in eight pairs of matched LSCC tissues; α-tubulin was used as the loading control. (**C**) Immunohistochemical analysis of CISD2 protein expression in the eight pairs of matched LSCC tissues, *P < 0.05.

To determine whether CISD2 is overexpressed in human LSCC, eight paired tumor samples (T) and the adjacent noncancerous tissues (ANT) from the same patients were subjected to quantitative real time PCR and Western blotting analyses. As illustrated in Figure 1A, CISD2 mRNA was significantly increased in LSCC tissue compared with the non-cancerous tissues. Consistent with the mRNA analysis, CISD2 protein was also over-expressed in LSCC tissues compared to the surrounding non-tumor regions (Figure 1B).

### Overexpression of CISD2 is associated with advanced clinical features in SCC

**Figure 2:**
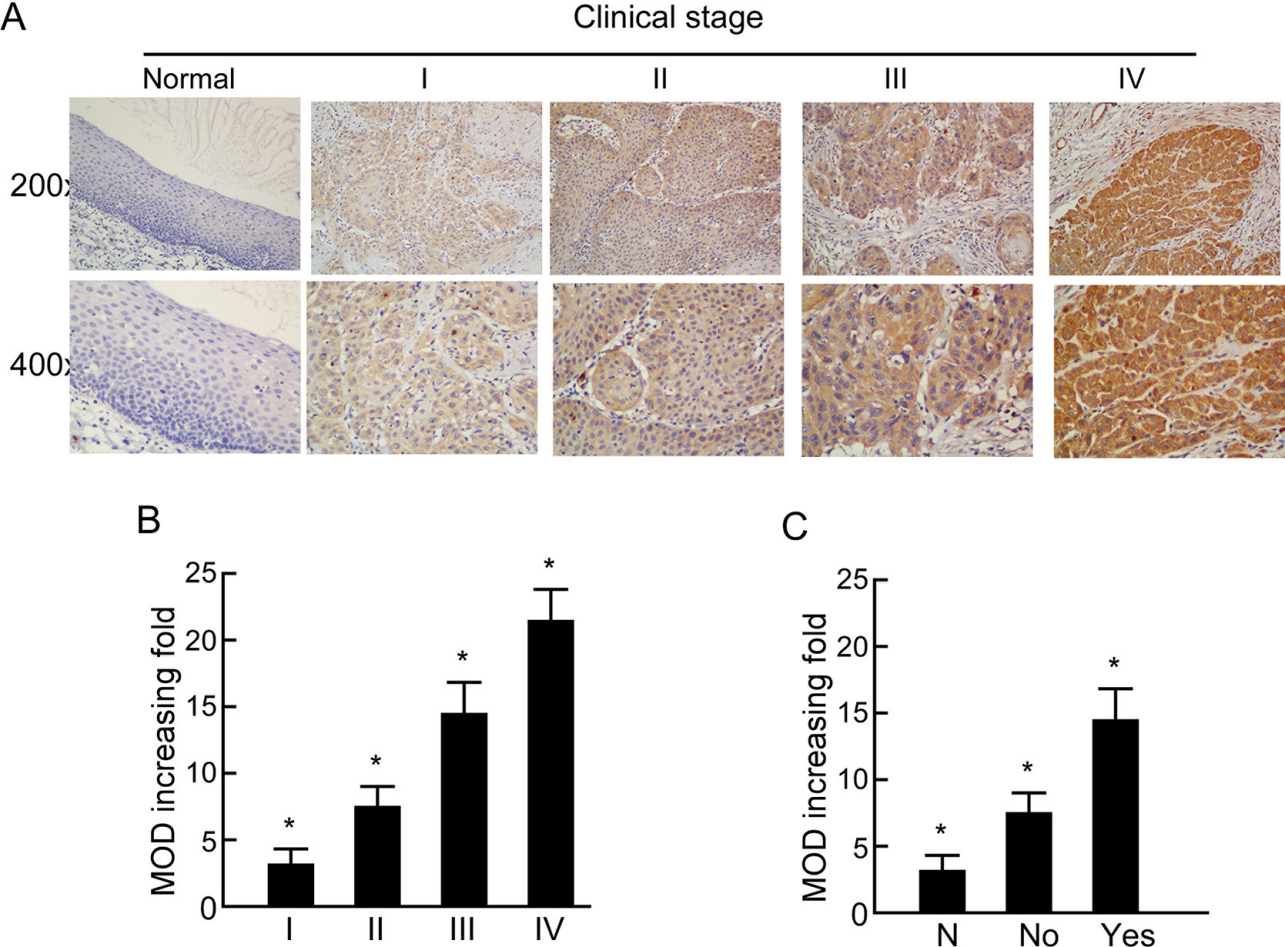
Expression of CISD2 in different clinical stages of laryngeal squamous cell carcinoma (LSCC) (**A**) Representative images of immunohistochemical staining for CISD2 in normal (control sections) LSCC tissues and different clinical stages of LSCC. (**B**) Average fold-change in the mean optical density (MOD) for CISD2 in different clinical stages of LSCC compared with normal laryngeal tissues. (**C**) The statistical analyses of the average MOD of CISD2 staining in the lymph node metastasis group and the lymph node metastasis-free group, *P < 0.05.

The positive CISD2 staining rate in the archived LSCC tissue was 95.4% (467/490). CISD2 was mainly localized to the tumor cell cytoplasmic with strong nuclei staining while little or no expression of CISD2 was observed in the normal epithelial cells (Figure 1C). In the training cohort, 118/245 (48.2%) cases were classified as high CISD2-expressing and 127/245 (51.8%) as low CISD2-expressing. Furthermore, CISD2 expression in LSCC samples increased with increasing clinical stage as shown by IHC staining intensity (Figure 2A). Quantitative IHC analysis revealed that the mean optical density (MOD) values of CISD2 staining in all of the SCC samples were higher than those in the normal control laryngeal tissues. Additionally, the MOD values of CISD2 staining significantly increased from stage I to IV (*P* < 0.05, Figure 2B). Moreover, the MOD values of CISD2 staining were markedly higher in the lymph node metastasis group than that in the lymph node metastasis free group (*P* < 0.001, Figure 2C).

We further analyzed the association between CISD2 and the clinicopathological characteristics of LSCC. There was no significant association between CISD2 expression and age, gender, smoking status, drinking status, pathological differentiation and tumor site. However, high CISD2 expression was significantly associated with advanced T stage (*P* < 0.001), N stage (*P* = 0.047) and clinical stage (*P* < 0.001), positive treatment method (*P* < 0.001) as well as risk of disease progression (*P* < 0.001) (Table 1). The results suggest that the CISD2 was a poor prognostic index of LSCC and higher CISD2 expression tend to be in a more advanced stage and receive more positive treatment methods compared with the lower CISD2 expression cohorts.

### Association between CISD2 expression and PFS in LSCC

**Figure 3:**
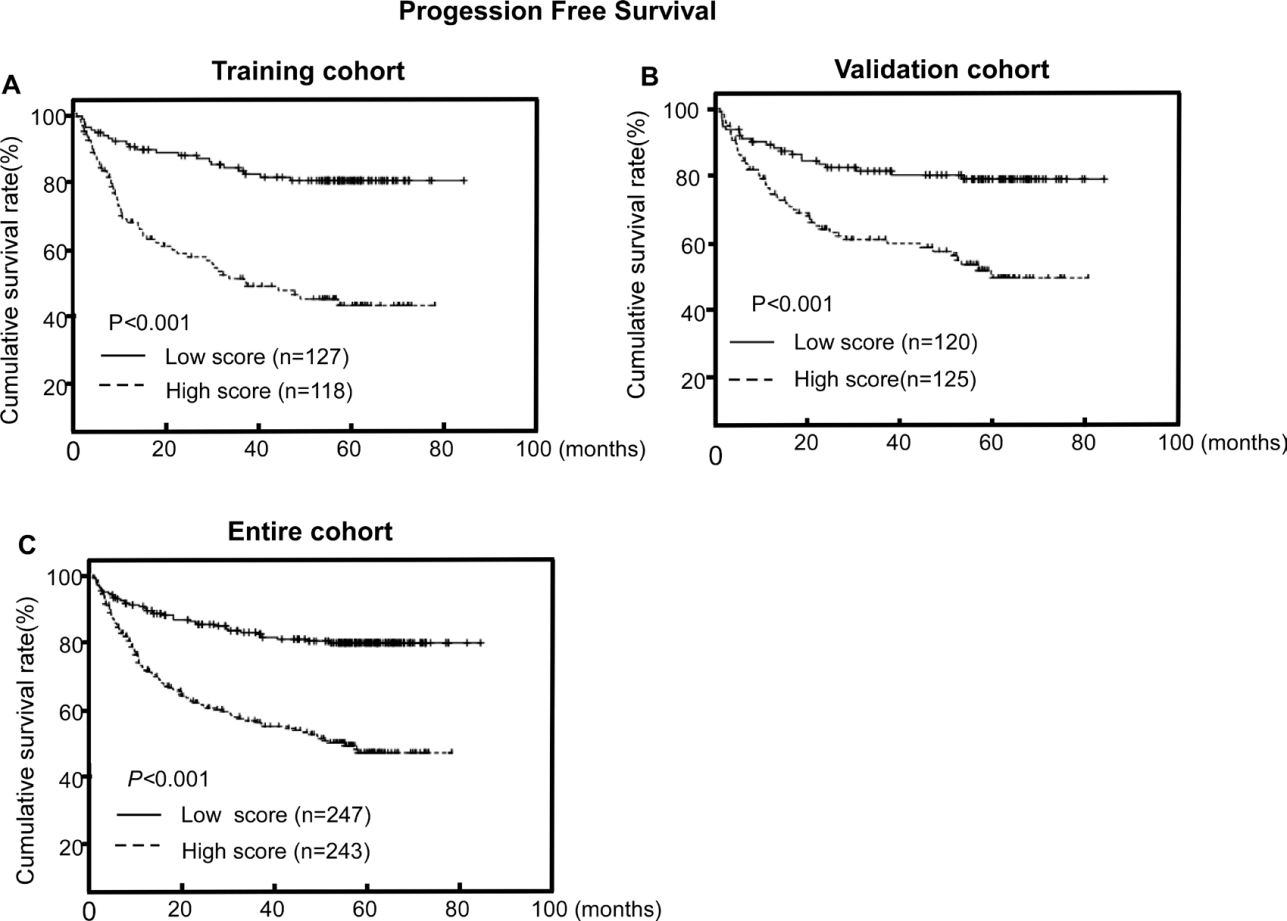
CISD2 protein expression is associated with progression-free survival (PFS) in LSCC (**A, B, C**) Kaplan–Meier PFS survival curves in the training cohort (A), validation cohort (B) and the entire cohort (C) stratified by high and low expression of CISD2. *P*-values were calculated using the log-rank test.

High CISD2 protein expression was significantly associated with poorer PFS in the training cohort, validation cohort and the entire cohort (*P* < 0.001, *P* < 0.001 and *P* < 0.001, respectively; Figure 3). The cumulative 5-year PFS rates for patients with lower CISD2 expression were 80.8% and 80.4% compared with that of 46.9% and 56.6% for patients with higher CISD2 expression in the training cohort and the validation cohort, respectively.

Univariate analysis showed that pathological differentiation (*P* = 0.023), tumor site (*P* = 0.047), T stage (*P* < 0.001), N stage (*P* < 0.001) and CISD2 expression (*P* < 0.001) were significant prognostic factors for PFS in LSCC (Table 2). Multivariate survival analysis was performed using the covariates that were significant in the univariate analysis. The results show that CISD2 and N classification remained independent prognostic factors for PFS (*P* < 0.001 and *P* < 0.001, respectively) (Table 2).

**Table 2:** Univariate and Multivariate Cox regression analysis of the association of various clinicopathological features with progression-free survival in training cohort

Feature	Univariate		Multivariate	
	Hazard ratio (95% CI)	*P*	Hazard ratio (95% CI)	*P*
Age (y) ≥ 45 vs < 45	1.213 (0.782–1.883)	0.388		
Gender F VSM	0.781 (0.109–5.613)	0.806		
Smoking status Present vs Absent	0.967 (0.498–1.876)	0.921		
Drinking status Present vs Absent	0.760 (0.478–1.208)	0.245		
Pathological differentiation		0.023		
Highly	1.000			
Moderately	1.767 (1.071–2.914)	0.026		
Poorly	2.253 (1.187–4.276)	0.013		
Site Non-glottic va Glottic	0.641 (0.412–0.995)	0.047		
T stage		< 0.001		
T1+T2	1.000			
T3	2.415 (1.427–4.087)	0.001		
T4	2.989 (1.744–5.125)	< 0.001		
N stage		< 0.001		< 0.001
N0	1.000		1.000	
N1	2.044 (1.163–3.592)	0.013	1.961 (1.115–3.451)	< 0.001
N2	3.862 (2.129–7.007)	< 0.001	3.027 (1.659–5.522)	0.019
CISd2 High vs Low	3.761 (2.280–6.203)	< 0.001	3.318 (2.030–5.425)	< 0.001

Abbreviations: SCC, laryngeal Squamous cell carcinoma (SCC); H, Highly differentiated; M, Moderately differentiated; P, Poorly differentiated; Non-Glottic, Supraglottic and Subglottic; Comprehensive, Surgery and chemotherapy or Surgery and radiotherapy; CISD2, CDGSH iron sulfur domain2.

### Risk score model for the PFS of LSCC

To create a more feasible score model for clinical practice, an integral score n value was assigned to each independent factor (called C-N model). The n value was calculated based on the hazard ratio (HR) of each independent risk factor: *n* = ln (HR) [16]. Subsequently, a score of 0, 2 or 3 was assigned to N classification and a score of 0 or 3 was assigned to CISD2 level (Table 3). The total scores ranged from 0 to 6 (0, 1, 2, 3, 5, 6) (mean = 1.97 and median = 3.00). The total score of the model is defined as. The cut-off values for CISD2 expression were chosen based on a measure of heterogeneity using the log-rank test with respect to progression-free survival (PFS). Therefore, the patients were divided into 3 risk subgroups based on their total score: low risk (score 0, 43.5%), middle risk (score 2–3, 40.8%) and high risk (scores 5–6, 15.7%).

**Table 3:** Calculation of the risk score and the C-N model for progression-free survival of the LSCC patients

Characteristic	*N* = ln HR	Score
N stage		
N0		0
N1	0.673	2
N2	1.108	3
CISD2 High vs Low	1.199	3
Low risk group		0
Middle risk group		2–3
High risk group		5–6

Abbreviations: LSCC, laryngeal Squamous cell carcinoma (LSCC); CISD2, CDGSH iron sulfur domain2; C-N model, CISD2 plus N stage model; HR, Hazard ratio

**Figure 4:**
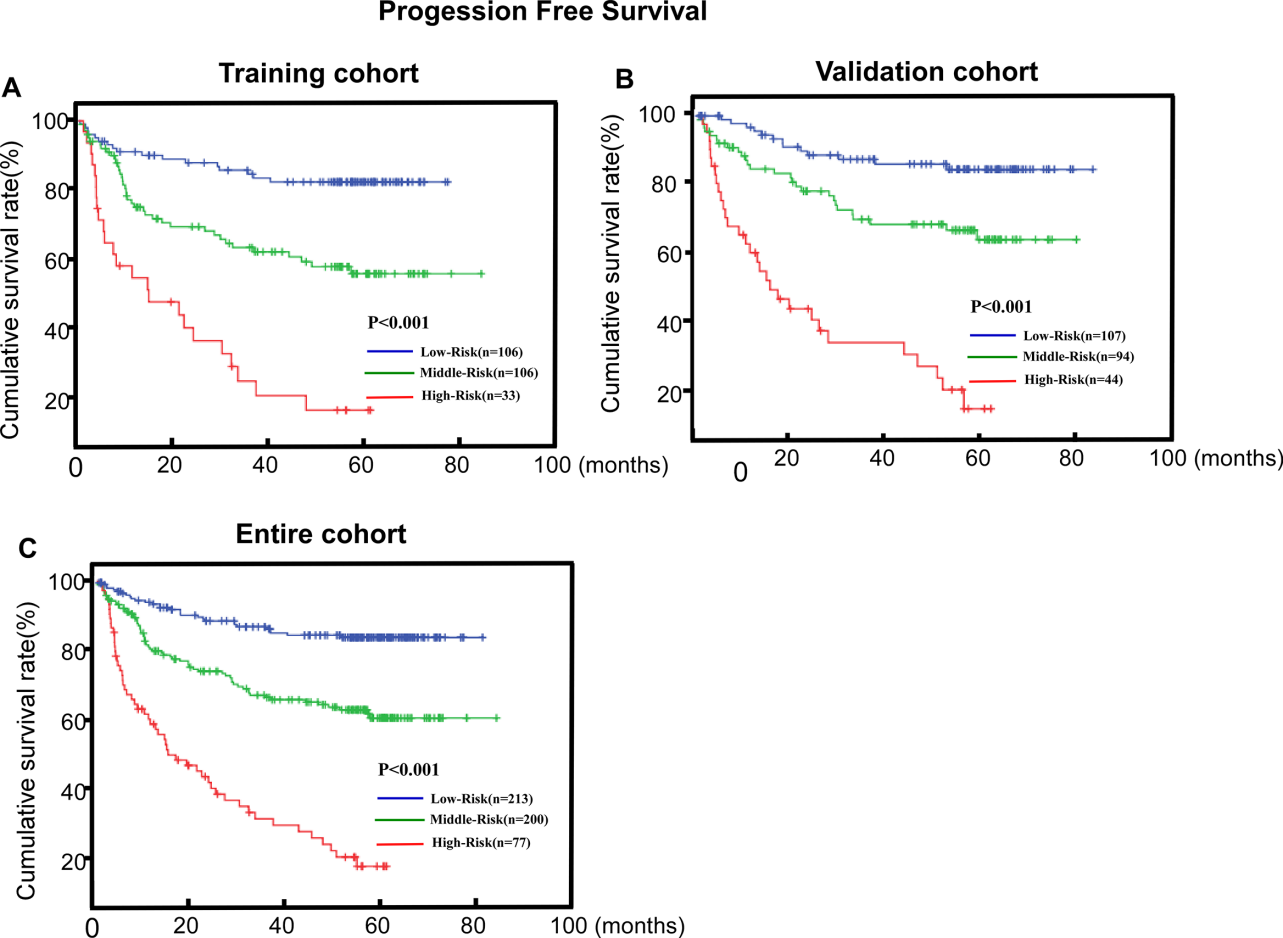
C-N model is associated with progression-free survival (PFS) in LSCC (**A, B, C**) Kaplan–Meier PFS survival curves in the training cohort (A), validation cohort (B) and the entire cohort (C) stratified by low, middle and high risk. *P*-values were calculated using the log-rank test.

The PFS curves discriminated significantly among the three risk subgroups in the training cohort, validation cohort and the entire cohort by the C-N model (*P* < 0.001, *P* < 0.001 and *P* < 0.001, respectively; Figure 4). The C-index of C-N model for predicting PFS in the training set and validation set were 0.710 (95% CI, 0.652–0.767) and 0.719 (95%CI, 0.663–0.774), which was significantly higher than that of TNM classification alone (0.602, 95% CI = 0.555-0.649, *P* = 0.027; 0.578, 95% CI = 0.517–0.640, *P* = 0.014). These results confirm that the C-N model was more precise in predicting PFS of LSCC patients than clinical classification was. In addition, the prognostic value of C-N model was mostly consistent across different TNM stage subgroups, with no identification of any interference (Table 4).

**Table 4:** Forest plot of subgroup effects for PFS in all LSCC patients

Characteristic	Subgroup of the Characteristic	Subgroup of the model	Hazard Ratio	95%CI		
				Low	High	*P*
Age	< 60	Middle vs Low	2.960	1.506	5.817	0.002
		High vs Low	7.542	3.699	15.379	< 0.001
	≥ 60	Middle vs Low	2.862	1.609	5.091	< 0.001
		High vs Low	10.945	6.028	19.872	< 0.001
Smoking status	Absent	Middle vs Low	1.616	0.567	4.609	0.369
		High vs Low	9.341	3.203	27.241	< 0.001
	Present	Middle vs Low	3.042	1.878	4.927	< 0.001
		High vs Low	8.637	5.214	14.308	< 0.001
Drinking status	Absent	Middle vs Low	3.024	1.789	5.113	< 0.001
		High vs Low	9.266	5.303	16.190	< 0.001
	Present	Middle vs Low	2.463	1.145	5.299	0.021
		High vs Low	7.599	3.479	16.600	< 0.001
Site	Glottic	Middle vs Low	3.191	1.458	6.984	0.004
		High vs Low	7.321	3.353	15.985	< 0.001
	Non-glottic	Middle vs Low	2.501	1.475	4.242	0.001
		High vs Low	12.002	6.605	21.812	< 0.001
T stage	I + II	Middle vs Low	2.771	1.558	4.928	0.001
		High vs Low	9.215	3.687	23.0333	< 0.001
	III + IV	Middle vs Low	2.509	1.217	5.173	0.013
		High vs Low	7.609	3.718	15.573	< 0.001
N stage	Negetive	Middle vs Low	2.974	1.915	4.619	< 0.001
		High vs Low	5.623	2.546	12.417	< 0.001
	Positive	Middle vs Low	3.149	1.739	5.705	< 0.001
		High vs Low	57.471	6.727	491.003	< 0.001
Clinical stage	I + II	Middle vs Low	2.205	1.106	4.396	0.025
		High vs Low	6.980	3.536	13.779	< 0.001
	III + IV	Middle vs Low	2.727	1.770	4.200	< 0.001
		High vs Low	8.349	5.318	13.105	< 0.001

Abbreviations: LSCC, laryngeal Squamous cell carcinoma (LSCC); PFS, Progression-free survival; Non-Glottic, Supraglottic and Subglottic; CISD2, CDGSH iron sulfur domain2; C-N model, CISD2 plus N stage model.

Subgroups are defined by factors showing significant associations between the C-N model and PFS.

## DISCUSSION

This is the first study to demonstrate that CISD2 over-expression is associated with poorer PFS in LSCC patients. The positive CISD2 staining rate in LSCC is as high as 95.4%, implying the critical role of CISD2 in the biological process of LSCC. Our study revealed that CISD2 is both transcriptionally and transnationally up-regulated in human LCSS. High CISD2 expression was significantly associated with unfavorable clinical features such as advanced T stage, N stage and clinical stage as well as disease progression. More importantly, high CISD2 expression is an independent predictor of poor PFS of LSCC.

In clinical practice, the prediction of risk of tumor progression after definite treatment is of great importance. An ideal prognostic model could aid clinician in choosing the optimal treatment strategies for patients, i.e. the application of adjuvant therapy, and to avoid over-treatment or insufficient treatment. However, due to the vast complexity of cancer, the development of satisfactory prognostic models is difficult and also imperative. For LSCC patients, a limited number of statistical prediction models have been proposed. *Tan et al.* have developed a nomogram utilizing age, hemoglobin, T stage and N stage at initial diagnosis to predict overall survival and local control rate of LSCC patients [17]. However, clinical classification alone is not perfect in predicting the survival of cancer patients due to the heterogeneity of clinical outcomes within equivalent classification. Till now, little prediction models have combined biomarkers and clinical characteristics for LSCC patients.

In the present study, we identify CISD2 level and N classification as independent prognostic factors for PFS in LSCC patients, which were used to build a novel prognostic model (C-N model). The C-N model successfully stratified LSCC patients into three risk subgroups and was more effective in predicting the prognosis of LSCC patients than clinical classification alone. Also, the prognostic value of this model was consistent across different clinical stages, with no identification of any interference. These results imply that CISD2 may represent a potential novel prognostic marker in LSCC and the C-N model could be applied in clinical practice to achieve a more accurate prognostication and facilitate the individualized treatment of LSCC patients in the future.

The treatment of LSCC patients need to consider the balance between sufficient treatment intensity and severe side effects. Previous studies have showed that surgery alone is associated with a high risk of loco-regional relapse and *Cooper et al.* had indicated that postoperative radiotherapy (PORT) plus systemic chemotherapy improves the clinical outcomes of high-risk HNSCC patients [3, 18, 19]. However, patients undergoing surgery plus chemotherapy or PORT experience higher frequency of severe side-effect such as such as mucositis, xerostomia, dysphagia, hematopoietic problems [20]. Until now, the identification of high-risk HNSCC patients remains a great challenge and reliable high-risk characteristics are lacking especially for early stage cancer patients. In the present study, we found that the C-N model could successfully stratify early stage LSCC patients into different PFS groups, implying that a subpopulation of early LSCC patients could be pulled out from the entire group for more intensive treatment. Further clinical studies, especially clinical trials are warranted to validate this hypothesis.

Lymphatic metastasis to the neck and lymphatic recurrence are the most common reasons of treatment failure for LSCC especially for the supraglottic carcinoma [21]. There is no controversy in neck dissection or radio-chemotherapy for patients with metastatic neck lymph nodes at initial diagnosis [22]. However, treatment strategies for patients with stage N0 are not consistent. Although simultaneous neck dissection is recommended for some N0 patients to prevent occult neck metastasis, no acknowledged standards are available to pick up the high-risk N0 patient [22]. In the present study, we found that high CISD2 expression was associated with cervical lymph node metastasis, consistent with a study in cervical cancer by *Liu et al.* [14]. More importantly, high risk C-N group in the negative lymph node metastases subgroup still have poorer PFS compare with low risk C-N group. Therefore, we suggest that this model might be useful for selecting high-risk patients from those without lymph node metastasis.

The potential link between high CISD2 expression and poorer PFS in LSCC might rely on the regulation of mitochondrial performance by CISD2 in cancer cells [23–25]. Some studies reported that mitochondrial homeostasis is essential to decide cell fate, such as cell proliferation, cell autophagy and death [26]. When this homeostasis is perturbed, usually by uncontrolled proliferation and a collateral failure to activate cell death, the susceptibility to cancer progression is inevitably increased [27]. Recent studies revealed that CISD2-BCL2 complex may negatively modulate the BECN1 autophagy-initiating complex via PIK3C3 and regulate endoplasmic reticulum (ER) Ca^2+^ homeostasis via ER inositol 1,4,5-triphosphate receptor (ITPR/IP3R) and contributes to tumor carcinogenesis and progression in LSCC [28]. More recently, *Tsai PH et al.* found that CSD2 deficiency impairs the activation of Wnt/β-catenin signaling, with the down-regulation of downstream genes, such as Tcf1, Fosl1, and Jun [29]. The Wnt/β-catenin signaling pathway has been confirmed as an essential player in the invasion and metastasis of LSCC and correlates with poor clinical prognosis [30]. The essential role of β-catenin in the process of metastasis has been highlighted in carcinomas of the head and neck [31]. Therefore, we hypothesize that CISD2, which is primarily located on the mitochondria, might also modulate the β-catenin in response to the activation of Wnt/β-catenin pathway to contribute to tumor carcinogenesis and progression in LSCC.

In conclusion, high CISD2 expression was associated with advanced clinical stage and might positively regulate LSCC development and progression. CISD2 may serve as a useful molecular biomarker of poor prognosis in LSCC. Clinical diagnosis in combination with assessment of CISD2 expression may improve stratification prognostication and help to identify high-risk patients with LSCC who may benefit from more aggressive treatment. However, the detailed molecular mechanisms of CISD2 in LSCC development and progression still require further investigation with. Of course, additional validation of the C-N model by prospective datasets could be useful. The main limitation of the manuscript is the lack of the cell-functional experiment to further demonstrate the mechanism of the association of the high-expression of the CISD2 and the poor prognosis in LSCC.

## MATERIALS AND METHODS

### Patients and tissue specimens

The enrollment criteria were as followings: 1) Patients were at stage I-IV without distant metastasis based on the TNM staging standard for laryngeal carcinoma established by UICC in 2002, and were treated primarily by surgery; 2) Patients were classified according to postoperative pathological TNM staging, the infiltration site from the primary locus and the lymphatic metastatic condition were confirmed using imaging examinations (ultrasound, CT or MRI); 3) Each patient had complete clinical and pathological data, including sex, age, smoking status, drinking status, pathological differentiation, tumor site, stage, treatment method, therapeutic effect, and recurrent condition; 4) patients with distant metastasis was exclude from the study. A total of 490 paraffin-embedded LSCC samples that were histologically diagnosed at Sun Yat-sen University Cancer Center (SYSUCC) between 2000 and 2009 were enrolled. The study was approved by the Institutional Research Ethics Committee of SYSUCC. Prior written patient consent was obtained for each patient. The clinical stages of all the patients were reclassified according to the 2002 UICC (Union for International Cancer Control) criteria. Clinical information pertaining to the samples is summarized in Table 1. All patients received standard treatment based on the clinical stages. In brief, patients with early-stage (stage I and II) tumors received surgery alone, whereas those with advanced-stage (stage III and IV) cancer received combination therapy comprising surgery and radiotherapy or chemotherapy [32]. The total irradiation dosage received by patients ranged from 66 to 80 Gy with a median dose of 70.0 Gy. All of the 490 surgical patients have undergone laryngectomy and neck dissection. 124 patients treated with chemotherapy as part of their primary treatment; 66 patients with cisplatin; 34 with cisplatin/docetaxe; 24 induction chemotherapy before radiotherapy or surgery.

### Follow up

Distant metastasis was evaluated by physical examination, head and neck magnetic resonance imaging (MRI), chest x-ray and/or CT, abdominal ultrasonography and bone scan every 6 months during the first three years after the completion of radiotherapy and annually thereafter. Survival follow-up was done via direct telecommunication or by referring to the clinic attendance records. The follow-up time ranged from 1.13–95.6 months with a median follow-up time of 55.1 months. A total of 153/490 (31.2%) patients experienced distant metastasis and/or recurrence during follow-up.

### RNA extraction and q-PCR

Total RNA was extracted from fresh LSCC tissues using TRIzol reagent (Invitrogen) per manufacturer's instructions. Total RNA was treated with RNase-free DNase and 2.0 μg of total RNA from each sample was subjected to cDNA synthesis using random hexamers. For PCR-mediated amplification of CISD2 cDNA, an initial amplification using CISD2-specific primers was performed with a denaturation step at 95°C for 10 min followed by 30 cycles of denaturation at 95°C for 60 s, primer annealing at 55°C for 30 s and extension at 72°C for 30 s. Upon completion of the cycling steps, a final extension was carried out at 72°C for 5 min before the reaction was stopped and stored at 4°. Quantitative real-time PCR was designed using the Primer Express v 2.0 software (Applied Biosystems) to determine the fold increases in CISD2 mRNA expression in the tumor specimens relative to noncancerous tissues. The sequences of the real-time PCR primers were: *CISD2* forward 5- GCAAGGTAGCCAAGAAGTGC-3 and reverse 5- CCCAGTCCCTGAAAGCATTA-3; *GAPDH* forward 5′- TTGAGGTCAATGAAGGGGTC-3′ and reverse 5′- GAAGGTGAAGGTCGGAGTCA-3′. *CISD2* expression data was normalized to *GAPDH*; all experiments were performed in triplicate.

### Western blotting

Fresh tissue samples were ground to powder in liquid nitrogen and lysed with SDS-PAGE sample buffer (62.5 mmol/L Tris-HCl pH 6.8, 2% SDS, 10% glycerol, and 5% 2-mercaptoethanol) and protein concentrations were determined using the Bradford assay (Bio-Rad Laboratories, Hercules, CA, USA). Equal protein samples (30 μg) were separated on 10.5% SDS polyacrylamide gels and transferred to PVDF (Polyvinylidene difluoride) membranes (Immobilon P, Millipore, Bedford, MA). Membranes were blocked with 5% fat-free milk in Tris-buffered saline containing 0.1% Tween-20 (TBST) for 1 h at room temperature, incubated with anti-CISD2 rabbit polyclonal antibody (1:1,000; Proteintech, City, Country) overnight at 4°C, then with horseradish peroxidase-conjugated goat anti-rabbit IgG (Santa Cruz Biotechnology, SC-2004), and CISD2 expression was detected using enhanced chemiluminescence system (ECL) prime Western blotting detection reagent (Amersham) according to the manufacturer's instructions. α-tubulin was used as a loading control.

### Immunohistochemistry

Immunohistochemistry was performed to assess CISD2 protein expression in the 490 human SCC tissues. In brief, 4 μm–thick paraffin-embedded sections were baked at 60°C for 2 h, followed by deparaffinized with xylenes and rehydrated, microwaved in EDTA antigen retrieval buffer. The sections were then treated with 3% hydrogen peroxide in methanol to quench endogenous peroxidase activity, incubated with 1% bovine serum albumin to block non-specific binding and incubated with an anti-CISD2 rabbit polyclonal antibody (1:50; Proteintech) at 4°C overnight. For negative controls, the primary antibody was replaced by normal goat serum. After washing, the tissue sections were treated with biotinylated anti-rabbit secondary antibody (Abcam), incubated with streptavidin horseradish peroxidase complex (Abcam). The tissue sections were immersed in 3-amino-9-ethyl carbazole, counterstained with 10% Mayer's hematoxylin, dehydrated and mounted in Crystal Mount.

The degree of immunostaining of the sections was scored by two independent observers who were blinded to the histopathologic features and patient data of the samples. The proportion of CISD2-expressing cells varied from 0% to 100% and the staining intensity varied from undetectable to strong. The intensity of staining were graded as 0 (no staining), 1 (weak, light yellow), 2 (moderate, yellowish brown) and 3 (strong, brown). The proportion of positive tumor cells was recorded as: 0 (no positive tumor cells), 1 (1%–10%), 2 (11%–35%), 3 (36%–70%) and 4 (> 70%). The staining index score (SIS) was calculated as staining intensity multiplying the proportion of positive cells for each section (potential scores: 0, 1, 2, 3, 4, 6, 9 or 12). The positive CISD2 staining is defined as the SIS 1, 2, 3, 4, 6, 9 or 12. The cut-off values for CISD2 expression were chosen based on a measure of heterogeneity using the log-rank test with respect to progression-free survival (PFS). Thus, the optimal cut-off for PFS was determined as: a SIS of > 6 as high CISD2 expression and a score of ≤ 4 as low CISD2 expression.

The method of mean optical density (MOD) was used to determine the immunostaining intensity of each tested specimen and was performed as previously reported [33]. In brief, the stained slides were evaluated at 200× magnification using the SAMBA 4000 computerized image analysis system with Immuno 4.0 quantitative program (Image Products International, Chantilly, Virginia). Ten representative staining fields of each tumor sample were analyzed to determine the Mean Optical Density (MOD), which represents the concentration of the stain as measured per positive pixels in the whole tissue.

A negative control with each batch of staining was used for background subtraction in the quantitative analysis. The MOD data were statistically analyzed using *t*-test to compare the average MOD difference between different group of tissues, *P* < 0.05 was considered significant.

### Statistical analyses

All statistical analyses were carried out using the SPSS version 20.0 statistical software packages. Pearson's *χ*^2^ and Fisher's exact tests were used to analyze the associations between CISD2 expression and clinicopathological features. Bivariate correlations between the study variables were calculated using Spearman's rank correlation coefficients. PFS was defined as the time from treatment initiation to the onset of recurrence, distant metastasis or the death because of LSCC confirmed by clinical assessment or MRI imaging. Survival curves were plotted using the Kaplan Meier method and compared with the log-rank test. Survival data were evaluated using univariate and multivariate Cox regression analyses. The regression coefficient of each independent variable was subsequently modified into an integer numerical value to simplify the computation [16]. A two-sided probability value < 0.05 was considered statistically significant. A total of 245 patients were randomly assigned to the training set to develop a prognostic score model. The remaining 245 patients were assigned to the validation of the prognostic model. The C-N model was subjected to 1000 bootstrap resamples for interval validation and external validation to correct the concordance index (c-index) and explain variance for over-optimism. The performance of the C-N model and TNM staging system for prediction survival were measured by c-index, an equivalent variable of the area under curve (AUC) of the receiver operating characteristic curve for censored data. The maximum value of c-index is 1.0 indicating a perfect prediction model while 0.5 indicates a random chance to correctly predict outcome by the model. Comparisons between C-N models and TNM staging were performed with the rcorrp.cens in Hmisc in R.
